# Semilunar line diastasis frequently coexists with rectus diastasis and may compromise outcomes after isolated midline repair

**DOI:** 10.3389/fsurg.2026.1895895

**Published:** 2026-08-07

**Authors:** Moshe Dudai, Marah Ganiem

**Affiliations:** Hernia Excellence, Tel Aviv, Israel

**Keywords:** abdominal wall asymmetry, abdominal wall reconstruction, core stability, hernia repair, rectus diastasis, semilunar line diastasis

## Abstract

**Background:**

Semilunar line diastasis (SLD) is a lateral fascial separation that may coexist with rectus diastasis (RD). Persistent lateral bulging and core instability after isolated RD repair suggested that unrecognized SLD may limit functional and aesthetic outcomes. This study characterizes SLD in patients with RD and reports early outcomes after concurrent repair.

**Methods:**

This single-center observational cohort included 13 consecutive adults with RD and SLD who underwent endoscopic abdominal wall reconstruction. The first four patients were operated on before SLD was recognized as a distinct clinical entity; two developed persistent lateral bulging, and two required secondary repairs. After introducing preoperative assessment of the semilunar lines, nine patients underwent simultaneous RD and bilateral SLD (BSLD) repair using a modified subcutaneous onlay laparoscopic approach (SCOLA), with midline and bilateral semilunar plication reinforced by wide midweight mesh. Outcomes included core stability, lateral contour, patient-reported aesthetic satisfaction, return to normal activity, and early complications.

**Results:**

Concomitant RD + BSLD repair was associated with improved core stability, correction of lateral bulging, high patient-reported aesthetic satisfaction and rapid return to normal activity. No major complications occurred; minor events, including seroma and superficial wound necrosis, resolved conservatively. Patients whose BSLD was not initially repaired had persistent lateral bulging or required secondary repair.

**Conclusion:**

In this preliminary descriptive series, SLD appeared to be clinically relevant in patients with RD. Concomitant repair of BSLD, when present, was associated with favorable functional and aesthetic outcomes. These findings support preoperative assessment of the semilunar lines and greater awareness of SLD during RD repair.

## Introduction

The anterior abdominal wall provides postural support, trunk stability, respiratory assistance, visceral protection and body contour. Its lateral integrity depends on the semilunar line—the tendinous junction between the rectus sheath and the lateral aponeuroses—which contributes to mechanical stability and symmetry of the lateral abdominal wall (1, 2). Disruption of this system can impair both function and appearance and may negatively affect quality of life.

Rectus diastasis (RD) is a midline separation of the rectus abdominis muscles along the linea alba. It is common in postpartum patients and in individuals with chronically elevated intra-abdominal pressure, and has been associated with both functional complaints and aesthetic concerns (3). RD has been formally defined and addressed by the European Hernia Society (EHS) guidelines, which provide diagnostic thresholds (including >2 cm separation), imaging-based assessment, and criteria for conservative vs. surgical management aimed at restoring the midline and abdominal wall stability (4).

In contrast, semilunar line diastasis (SLD) refers to separation or attenuation at the semilunar line lateral to the rectus muscles, but remains largely unrecognised and is not included in current classifications or guidelines. SLD may present with lateral abdominal bulging, pain, core instability, functional limitation in daily activity, and visible asymmetry, as observed in our series (5). If left unaddressed, SLD may progress toward true lateral herniation with potential for incarceration or strangulation, a risk anatomically related to the semilunar region and to the site of Spigelian hernias (6).

Given the limited recognition of semilunar line diastasis and its potential impact on surgical outcomes, this study aimed to characterise SLD in patients with RD, report early functional and aesthetic outcomes following a standardized surgical approach, and draw surgeons' attention to the need to assess for SLD when planning RD repair.

## Methods

### Study design

This study was a retrospective observational analysis based on data collected during routine clinical practice at a private medical center (Ramat Aviv Medical Center, Tel Aviv, Israel). All procedures were performed by a single surgeon between 2019 and 2024.‏ No additional intervention was performed solely for research purposes. According to institutional policy for retrospective analyses of routine clinical data, the requirement for formal ethics committee approval was waived. All patients provided informed consent before surgery, including consent for the use of anonymised clinical data and clinical images for research and publication purposes. This study is reported in accordance with the Strengthening the Reporting of Observational Studies in Epidemiology (STROBE) guidelines.

### Patient selection and evolution of the diagnostic and operative strategy

The cohort comprised 13 consecutive adults who underwent surgical repair of RD between 2019 and 2024 and in whom SLD was identified either during preoperative assessment or, in the early cases, during postoperative follow-up. Patients were identified from routine clinical records. SLD ascertainment was based on clinical examination, but primarily on cross-sectional imaging, as described below. No additional exclusion criteria were applied. Postoperative follow-up was conducted as part of routine clinical care, and postoperative cross-sectional imaging was obtained when clinically indicated rather than according to a standardized study protocol.

The diagnostic and operative strategy evolved sequentially during the study period and was not based on a prospectively defined comparative protocol. Cases 1 and 2 underwent isolated RD repair before routine assessment of the semilunar lines had been introduced. Cases 3 and 4 also initially underwent isolated RD repair and were subsequently diagnosed with bilateral semilunar line diastasis (BSLD) on clinical and radiological follow-up; both later underwent planned second-stage BSLD repair.

These early observations prompted the introduction of systematic preoperative clinical and radiological assessment of the semilunar lines in subsequent patients undergoing RD repair. When SLD was identified, patients were counselled regarding its uncertain clinical significance and the rationale for concomitant repair. Cases 5–13 subsequently underwent simultaneous RD and BSLD repair as part of individualized surgical planning after providing informed consent.

**Figure 1:**
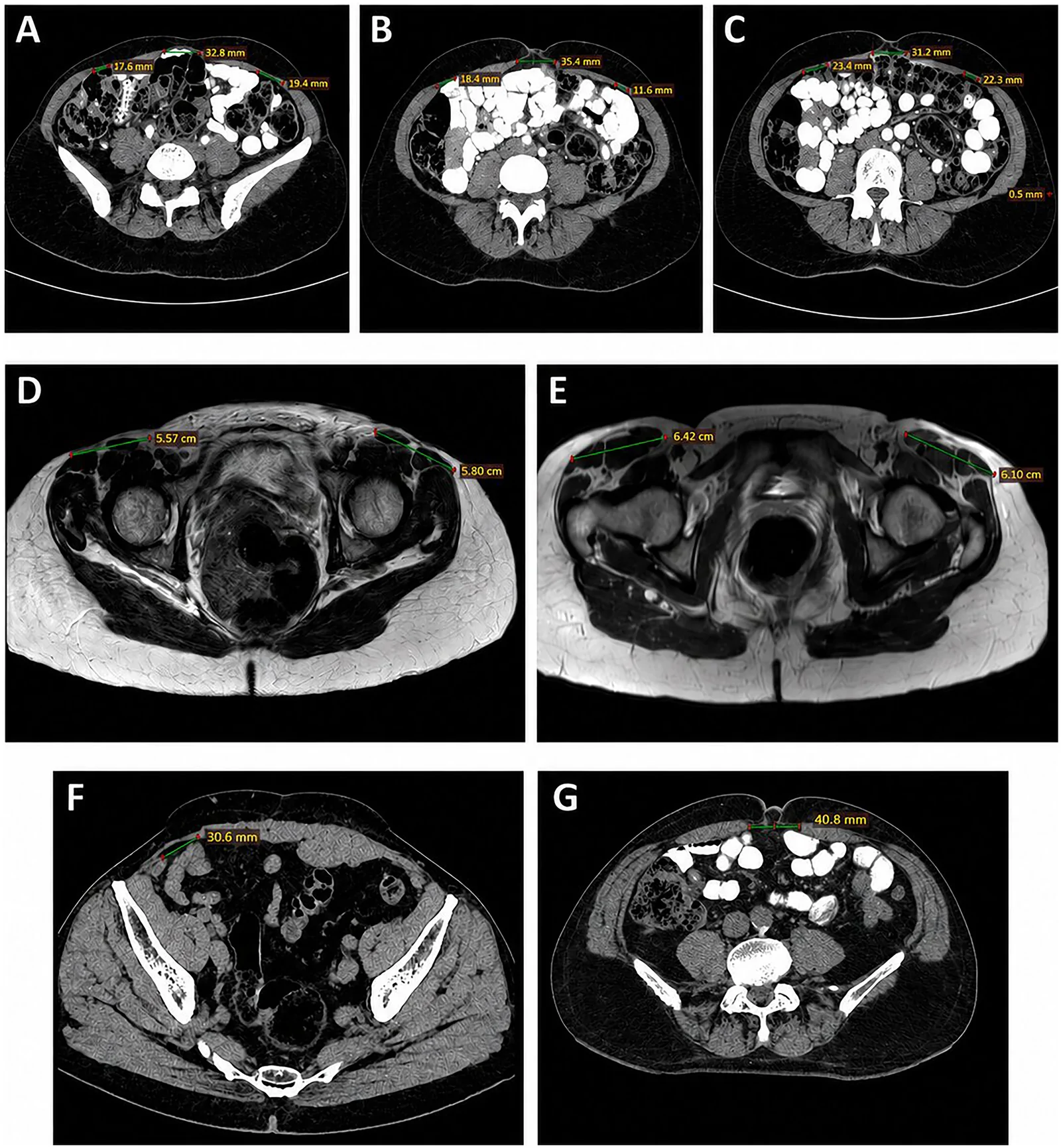
Representative pre- and postoperative cross-sectional imaging from two early cases treated with isolated midline RD repair. **(A–C)** Preoperative axial CT images from Case 1 at three abdominal levels, demonstrating RD with minimal BSLD. **(D,E)** Postoperative axial MRI images from the same patient, demonstrating marked bilateral SLD on follow-up imaging. **(F,G)** Pre- and postoperative axial CT images from Case 2, respectively, demonstrating an increase in right-sided SLD from 1.3 cm preoperatively to 3.6 cm postoperatively following RD repair, without a comparable increase on the left. The overlaid calipers indicate the measured RD and SLD widths at the respective axial levels.

Treatment allocation was neither randomized nor governed by a prespecified comparative protocol; rather, it reflected the chronological evolution of the clinical recognition and management of SLD during the study period. Accordingly, patients treated during the early and later phases of the series were regarded as descriptive clinical cohorts rather than prospectively defined comparison groups. Representative imaging from two early cases that contributed to this change in clinical practice is shown in Figure 1.

### Definitions of conditions and measurements

RD was defined as separation of the rectus abdominis muscles along the Linea alba greater than 2 cm on clinical or radiological assessment, in line with EHS recommendations (4).

SLD was defined as separation or attenuation of the semilunar line in the absence of a discrete full-thickness fascial defect or hernia sac. It was suspected clinically based on lateral abdominal wall bulging and/or asymmetry and confirmed on CT by visible widening of the semilunar line. The absence of a focal fascial defect or hernia sac distinguished SLD from a Spigelian hernia. BSLD was defined as involvement of both semilunar lines. Patients with BSLD typically presented with a “frog belly” appearance, characterized by broad bilateral lateral abdominal wall bulging, widening of the abdominal contour, and asymmetry caused by unequal prominence of the two sides. In the absence of validated diagnostic criteria for SLD, the >2 cm threshold was adopted provisionally from EHS criteria for RD (4).

RD and SLD widths were measured on axial CT images obtained during Valsalva. The reported measurement referred to the widest level of separation, with maximal RD width measured at the linea alba and right and left SLD widths measured at the widest axial level. Preoperative CT-based measurements were confirmed intraoperatively.

For exploratory descriptive purposes, the authors developed a non-validated five-component composite core-instability score. The components comprised lower back pain, abdominal wall pain, pelvic floor dysfunction, impaired abdominal muscle function, and disturbed core stability. These components were selected based on symptoms commonly reported in studies evaluating functional outcomes after RD repair (7).

For exploratory descriptive purposes, the authors developed a non-validated composite core-instability score based on five functional domains described in the European Hernia Society guidelines on rectus diastasis: lower back pain, abdominal wall pain, pelvic floor dysfunction, impaired abdominal muscle function, and disturbed core stability (4, 7). The EHS guidelines describe these clinical domains but do not provide a scoring system. The numerical scoring method used in the present study was therefore developed by the authors specifically for this survey and analysis. This author-developed exploratory measure has not been validated as a patient-reported outcome instrument. Each patient received a score ranging from 0 to 5 according to the number of components identified preoperatively.

Functional and aesthetic improvement was assessed using an adapted patient-reported Global Rating of Change-type scale ranging from −3 to +3. Negative values indicated worsening, 0 indicated no change, and positive values indicated improvement (8). Return to normal activity was defined as the number of days until patients resumed daily routines without restrictions.

### Surgical technique

**Figure 2:**
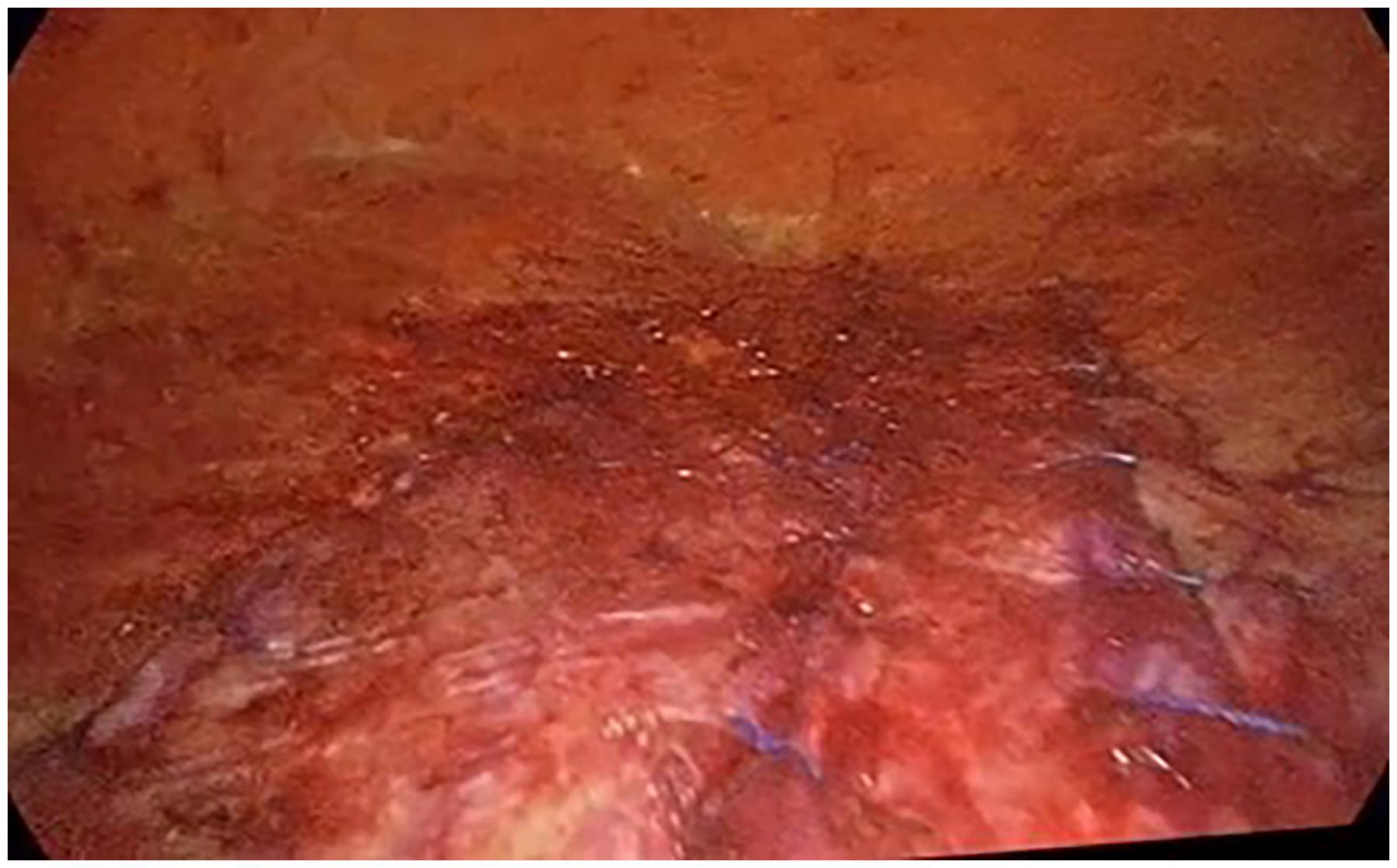
Three plication lines of RD and bilateral SLD.

All procedures were performed using a modified Subcutaneous Onlay Laparoscopic Approach (SCOLA) technique. In the lithotomy position, using three small pubic ports, a subcutaneous plane was developed over the anterior rectus sheath from the pubis to the xiphoid process. For midline repair, the complete linea alba was plicated using running non absorbable sutures, and a second overlapping plication of the anterior rectus fascia was performed over the first. In selected cases with increased tension, Rectus Fascia Release (RFR) was performed as a tension-reducing manoeuvre by making bilateral longitudinal incisions in the anterior rectus sheath with an electrosurgical hook, approximately 4 cm lateral to the medial border of each rectus muscle and extending 5 cm above and below the umbilicus. This adjunct mobilises and relaxes the rectus fascia and is conceptually related to anterior component separation, although it does not involve muscle division or incision at the semilunar line. In cases where SLD was recognised, bilateral plication of the semilunar lines was performed with running sutures back and forth along the lateral fascial edges to restore continuity. A wide midweight mesh (approximately 17–18 cm) was placed in the onlay position to reinforce both the midline and the lateral repair and was fixed using biological glue (Figure 2). In the first two patients, only midline plication with mesh reinforcement was performed. Patients 3 and 4 later underwent a secondary operation for BSLD repair after postoperative identification of persistent lateral bulging. In the remaining nine patients, semilunar line repair was performed concurrently with the midline repair according to the updated protocol.

Dead-space management and seroma prevention evolved during the study period. Since 2019, intraoperative hypertonic saline irrigation/injection (IHSI) has been used as part of the SCOLA protocol to reduce seroma formation after wide subcutaneous dissection (9). From 2022 onward, this approach was refined into the TASP protocol, which combines quilting sutures to reduce subcutaneous dead space, 12% hypertonic saline irrigation/injection, and final obliteration of the dissected space with biological glue.

### Outcomes

The primary outcomes were postoperative author-developed, nonvalidated core stability score (assessed by change in the number of instability components) and patient-reported aesthetic satisfaction. Secondary outcomes included time to return to normal activity and postoperative complications such as seroma or wound necrosis. Functional ability during activities such as walking, standing, lifting and sports was assessed qualitatively through patient interviews.

### Follow-up and data collection

Patients were evaluated clinically at routine postoperative visits. Standard follow-up included photographic documentation before surgery and at 3–4 months after surgery to assess abdominal contour. At 3 months after surgery, patients were referred for a 3-month course of physiotherapy. At the 6-month postoperative assessment, patients rated functional and aesthetic changes using the patient-reported −3 to +3 scale. The 6-month assessment was used as the common follow-up time point for the outcomes reported in this study. Time to return to normal activity and postoperative complications were obtained from the clinical follow-up records. Any later recurrence information was derived from routine clinical follow-up rather than from a standardized long-term assessment protocol.

### Bias and limitations in data capture

This was a retrospective observational series. All operations and postoperative assessments were performed by a single surgeon, introducing potential observer and performance bias. The operative protocol evolved over time: in the initial patients only RD was repaired, whereas in later patients both RD and BSLD were repaired concurrently once SLD was systematically identified preoperatively. This change created two clinically distinct sequential groups (isolated midline repair vs. combined midline and semilunar line repair) and enabled descriptive comparison of outcomes between approaches. Because allocation to each approach was sequential rather than prospective or randomized, observed differences between groups may also reflect temporal or selection effects in addition to the surgical technique itself.

### Statistical analysis

Data were summarized using means and ranges. Because of the small cohort and observational design. Descriptive comparisons were made between patients who received midline repair alone and those who underwent combined midline and semilunar repair.

### Ethics and consent

This retrospective observational study used data collected during routine clinical care. Under institutional policy, formal ethics committee approval was waived because the study involved retrospective analysis of clinical data and did not involve any study-specific intervention or additional patient risk. All patients provided informed consent before surgery, including consent for the use of clinical data and clinical images for research and publication purposes.

## Results

### Patient cohort

Thirteen patients (seven women and six men) with a mean age of 51 years (range 34–73) were included. All 13 patients completed the 6-month postoperative assessment. The median follow-up duration for the reported outcomes was 6 months (range, 6–6 months).

### Preoperative findings

Preoperative RD widths ranged from 4 to 7 cm. In Cases 1–4, borderline semilunar separation on preoperative CT was noted (∼2 cm) but was not considered significant at the time of surgery. After midline plication, postoperative CT revealed that semilunar separation increased to approximately 3.6–5.5 cm in these cases. In Cases 1–2, patients underwent midline repair only, both subsequently developed significant lateral bulging. In Cases 3–4, BSLD was recognized postoperatively, and a second procedure to repair the semilunar lines was performed. Cases 5–13 underwent simultaneous midline and semilunar repair at the initial operation. Preoperative SLD widths ranged from 1.4 to 4 cm and were typically wider on the right side. Associated hernias included umbilical hernias (Cases 1, 4 and 13), an epigastric hernia (Case 4) and a postoperative incisional hernia (POIH) (Case 13). Patients' characteristics and preoperative clinical findings are summarized in Table 1.

**Table 1:** Pre-operative findings.

Case num	Gender	Age	RD (cm)	SLD (cm)		Abdominal wall asymmetry (right/left)	Umbilical hernia (cm)	EPI Hernia
				Right	Left			
1^a^	Female	44	6	Pre op 2	Pre op 3	Right	2	
				Post op 4.8	Post op 5.4			
2^a^	Male	66	4	Pre op 2	Pre op 2	Right	2	
				Post op 3.8	Post op 3.6			
3^b^	Female	34	5	Post Op 7	Post Op 7	Right	2	
4^b^	Male	51	6	Post Op 4	Post Op 3	Right	3	3 (recurrent)
5	Male	67	6 (recurrent)	4	3	Left		
6	Male	38	4.5	2	3	Right	5	
7	Male	55	4.5	2	No	Right	1.5	
8	Female	41	5.5	2.5	1.5	Right	2.2	
9	Male	52	4.5	3	3	Right	3.5	
10	Female	73	5.5	3	3.5	Right	2	
11	Female	43	7	3.5	3	Right	3	
12	Female	39	4.5	2.5	2	Right	2	
13	Female	60	5.6	2	1.4	Right	2	POIH 4X2 cm

a SLD was not required.

b SLD repaired in a second stage surgery.

Postoperative findings are summarized in Table 2. At the 6-month clinical assessment, Case 1 experienced postoperative worsening in core stability, functionality, and aesthetics. Cases 2 and 3 had no preoperative core instability. However, Case 2 showed postoperative deterioration in functionality and aesthetics. In contrast, patients in the combined repair cohort reported marked improvement across all evaluated domains. Among those who underwent revision surgery for BSLD after initial midline repair (Cases 3–4) and those who underwent simultaneous RD and BSLD repair (Cases 5–13), substantial improvement was consistently observed. Of these 11 patients, nine rated their functional outcome as +3, and two as +2. Aesthetic satisfaction scores were similarly high, with eight patients rating +3 and three rating +2.

**Table 2:** Postoperative results.

Case num	Return to normal activity (days)	Seroma	Other complication	Preoperative core instability (0–5)	Postoperative core instability improvement	6 Months status (−3 to +3)	
						Function	Aesthetic
1^a^	6	No	No	3	Same	(−) 1 worsening	(−) 3 worsening
2^a^	6	Small	Old scar necrosis	0	No	(−) 2 worsening	(−) 3 worsening
3^b^	5	No	No	0	No	3	3
4^b^	5	No	No	2	Improved	3	3
5	7	No	No	2	Improved	3	2
6	3	No	No	2	Improved	3	3
7	6	No	No	3	Improved	3	2
8	5	No	No	4	Improved	3	3
9	7	Yes	No	5	Improved	2	2
10	5	No	No	3	Improved	3	3
11	7	No	No	3	Improved	2	3
12	5	No	No	4	Improved	3	3
13	7	No	No	1	Improved	3	3

a SLD was not required.

b SLD repaired in second stage surgery.

Return to normal activity occurred within 3–7 days for all patients. Two patients (Cases 2 and 9) developed postoperative seromas that resolved conservatively without intervention, and one patient (Case 2) experienced superficial wound necrosis of a pre-existing scar that healed with conservative management. No recurrences or major complications were documented by the 6-month postoperative assessment.

**Figure 3:**
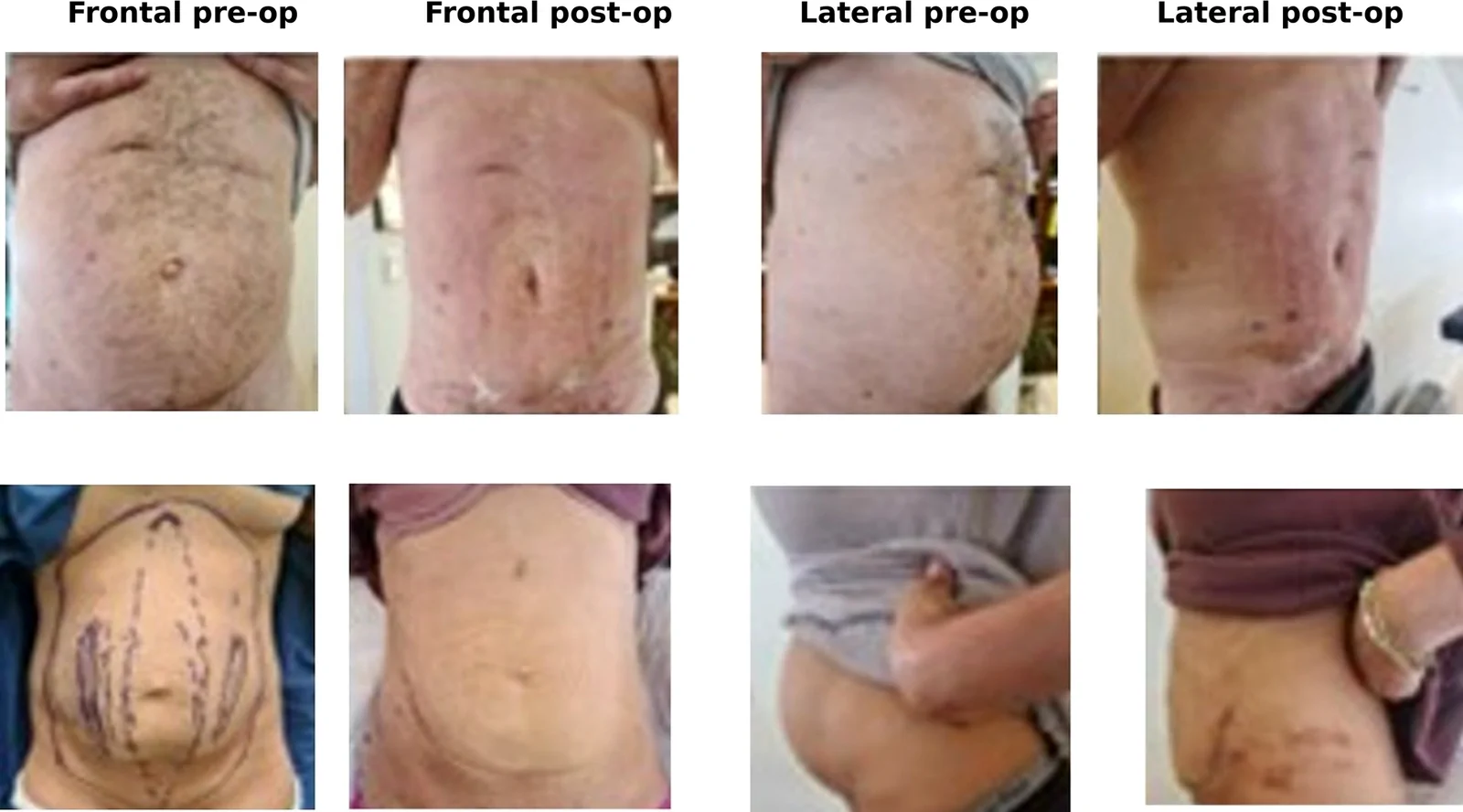
Pre- and postoperative clinical images of male (top) and female (bottom) patients. Each row demonstrates frontal preoperative, frontal postoperative, lateral preoperative, and lateral postoperative views. Postoperative images show resolution of lateral abdominal bulging and abdominal-wall asymmetry, described in this report as the ‘frog belly’ appearance, following simultaneous repair of RD and BSLD using modified SCOLA.

In this descriptive series, patients treated after implementation of concomitant RD and BSLD repair showed improvement in abdominal contour and patient-reported functional and aesthetic outcomes, whereas early patients treated before recognition of SLD experienced persistent or progressive lateral bulging, poor aesthetic satisfaction and no improvement in core instability. Figure 3 illustrates representative pre- and postoperative clinical images demonstrating improvement in abdominal contour and symmetry following simultaneous repair of RD and BSLD in male and female patients.

## Discussion

This observational study suggests that SLD may represent an under-recognized contributor to abdominal wall dysfunction and cosmetic dissatisfaction in some patients undergoing RD repair. In the early cases in this series, isolated midline repair was followed by postoperative widening of the semilunar lines. These observations raise the hypothesis that midline plication may expose or exacerbate pre-existing semilunar weakness when SLD is already present but remains unrecognized or is considered clinically insignificant before surgery. Conversely, simultaneous repair of RD and BSLD using a modified endoscopic SCOLA technique was associated with favorable early functional and aesthetic outcomes and few complications. These preliminary findings support consideration of semilunar-line assessment during preoperative planning.

The semilunar line marks the interface between the rectus abdominis and the lateral abdominal muscles. It is biomechanically plausible that attenuation or separation in this region could reduce lateral abdominal-wall support and permit displacement of the abdominal contour. We hypothesize that midline plication concentrates tensile forces centrally, which could redirect intra-abdominal pressure laterally, widening an unrecognized semilunar defect. Bilateral plication of the semilunar line defects restores continuity of the lateral fascial compartment, redistributes forces across the abdominal wall, and may improve core stability. This rationale aligns with biomechanical principles of fascial reconstruction and supports the observed clinical improvements (10–12). However, these proposed mechanisms were not directly tested in the present study.

Previous work by Medina et al. (13) and Dudai (14) described SCOLA, a fully endoscopic subcutaneous approach for the repair of RD and other midline abdominal wall defects. In the present series, the technique was modified by extending the dissection and plication laterally to include both semilunar lines, thereby enabling simultaneous repair of RD and BSLD in a single procedure. Incorporating semilunar-line repair into endoscopic approaches such as SCOLA may help address residual lateral bulging and asymmetry and, consequently, improve patient satisfaction and quality of life after abdominal wall reconstruction.

Preoperative assessment of the semilunar lines may be considered in patients undergoing RD repair. When BSLD is identified or suspected, simultaneous repair of the midline and lateral defects may require extension of the operative field, bilateral semilunar-line plication, and reinforcement with an appropriately sized mesh. Preoperative counselling should address the uncertain clinical significance of SLD, the potential for persistent lateral bulging and core instability if the lateral defects remain untreated, and the expected adaptation of the overlying skin and subcutaneous tissues after restoration of the abdominal contour. Although this adaptation was generally satisfactory in the present series, it may be less complete in patients with substantial pre-existing skin and adipose-tissue redundancy.

This study has several important limitations. (I) It is a small retrospective descriptive series of 13 patients and was not powered for inferential analysis.‏ Therefore, no causal conclusions can be drawn regarding the independent effect of SLD repair. (II) All procedures were performed by a single surgeon, limiting generalizability and introducing potential observer and performance bias. (III) The >2-cm diagnostic threshold was provisionally adopted from EHS criteria for RD because validated diagnostic criteria for SLD are lacking. (IV) Outcomes were assessed primarily through patient reports, clinical examination, and photographic documentation and were therefore subjective. (V) The core-instability score was an author-developed exploratory measure and has not been validated as a patient-reported outcome instrument. (VI) The common follow-up period for the reported outcomes was limited to 6 months. These findings should therefore be regarded as preliminary and require confirmation in larger prospective multicenter studies with predefined diagnostic criteria, standardized treatment protocols, and validated long-term outcome measures.

In conclusion, in this preliminary retrospective series, simultaneous repair of RD and BSLD using a modified endoscopic SCOLA technique appeared feasible and was associated with favorable early functional and aesthetic outcomes. Failure to recognize BSLD may contribute to persistent lateral bulging, asymmetry, and patient dissatisfaction after isolated midline repair. Preoperative assessment of the semilunar lines should be considered in patients undergoing RD repair, and concomitant repair of midline and lateral defects may be appropriate when BSLD is clinically and radiologically identified. These preliminary observations require confirmation in larger prospective studies.

## Data Availability

The raw data supporting the conclusions of this article will be made available by the authors, without undue reservation.
